# A Critical Review of Studies Assessing Interpretation Bias Towards Social Stimuli in People With Eating Disorders and the Development and Pilot Testing of Novel Stimuli for a Cognitive Bias Modification Training

**DOI:** 10.3389/fpsyg.2020.538527

**Published:** 2020-09-29

**Authors:** Katie Rowlands, Emma Wilson, Mima Simic, Amy Harrison, Valentina Cardi

**Affiliations:** ^1^Department of Psychological Medicine, Institute of Psychiatry, Psychology and Neuroscience, King’s College London, London, United Kingdom; ^2^Department of Psychology, Institute of Psychiatry, Psychology and Neuroscience, King’s College London, London, United Kingdom; ^3^Child and Adolescent Eating Disorders Service, South London and Maudsley NHS Foundation Trust, London, United Kingdom; ^4^Department of Psychology and Human Development, University College London, London, United Kingdom; ^5^Department of General Psychology, University of Padova, Padova, Italy

**Keywords:** interpretation bias, eating disorders, social, interpersonal, cognitive bias modification

## Abstract

People with eating disorders display a negative interpretation bias towards ambiguous social stimuli. This bias may be particularly relevant to young people with the illness due to the developmental salience of social acceptance and rejection. The overall aim of this study was to systematically develop and validate stimuli for a cognitive bias modification training to reduce a social rejection-related negative interpretation bias in young people with eating disorders. A mixed-methods design was used to achieve this aim. A review of the literature was conducted using EMBASE, MEDLINE, PsycINFO, Web of Science, and PubMed. Six studies were included in the review. Focus groups were held with patients with eating disorders, carers and healthcare professionals. Content analysis was used to identify key themes from the qualitative data. Based on these themes, a total of 339 scenarios were generated by the researchers. Salient themes identified from the focus group data included virtual rejection/exclusion, rejection associated with an aspect of the eating disorder, rejection triggered by ambiguous/benign comments or behaviors of others and rejection perceived when confiding in others. Patients rated these scenarios in terms of their age-relevance and emotional salience and 301 scenarios were included in the final stimulus set. These materials may be used by researchers conducting future experimental research into the potential benefits of interpretation bias training for young people with eating disorders.

## Introduction

Mental health disorders place a huge burden on the individuals affected, their families and society (Holmes et al., 2018). Despite the availability of evidence-based psychological treatments, their efficacy is sub-optimal and requires coordinated efforts from researchers and clinicians worldwide to improve (Holmes et al., 2018). The use of evidence-based practice is key to treatment innovation. Evidence-based practice refers to treatment guided by a combined consideration of clinical expertise, research evidence and patient values, preferences and circumstances (American Psychological Association, 2005). Recently, it has been suggested that this approach is particularly suited to improve treatment for conditions characterized by repeated treatment failures and chronicity, such as eating disorders (Peterson et al., 2016; Hilbert et al., 2017). In this study, we will use the evidence-based practice framework to validate the materials for a novel computerized training to improve social functioning in young people with eating disorders.

Eating disorders are psychiatric conditions diagnostically characterized by abnormal eating behaviors and cognitions related to eating, weight and shape (American Psychiatric Association, 2013). These symptoms often appear in adolescence, a time when the pressure for social acceptance is critically high (Somerville, 2013). Among other factors, the onset of abnormal eating behaviors in adolescence has been associated with criticism from others with regards to general aspects of the self, eating, and physical appearance (Jacobi et al., 2011; Copeland et al., 2015; Duarte et al., 2017; Lee and Vaillancourt, 2019; Lie et al., 2019; Okada et al., 2019). Furthermore, the severity of eating disorder symptoms has been linked to greater concerns about appearing nervous or anxious to others (Espel-Huynh et al., 2019). Experimental studies have corroborated the causal role played by interpersonal stress in triggering eating disorder symptoms (Cardi et al., 2018b; Monteleone et al., 2018). For instance, tasks that elicit interpersonal stress increase the desire to binge eat in patients with bulimia nervosa or binge eating disorder (Tuschen-Caffier and Vogele, 1999; Hilbert et al., 2011; Rosenberg et al., 2013), and reduce the liking of food in women with a lifetime diagnosis of anorexia nervosa compared to healthy women (Chami et al., in preparation). The implication of these findings is that decreasing reactivity to interpersonal stress might reduce the severity of eating disorder symptoms.

Recent advances in the field of experimental psychopathology indicate that it is possible to lower patients’ sensitivity to negative social feedback by reducing their tendency to interpret ambiguous social information in a negative way (Cardi et al., 2015, 2019; Turton et al., 2018).

Cognitive Bias Modification for Interpretation (CBM-I) is a computerized training developed with the goal of reducing a negative interpretation bias by exposing participants to benign/neutral interpretations of ambiguous social scenarios. Both adults and adolescents with eating disorders display an interpretation bias towards negative social information and this bias is related to self-reported sensitivity to rejection as well as core eating disorder symptoms, such as fear of weight gain and body image disturbance (Cardi et al., 2017). A recent study also indicated that cognitive biases (attention and interpretation biases) towards negative or ambiguous social information are malleable to change after using five training sessions of combined CBM-I and CBM of attention (CBM-A) and that the use of the training is associated with lower levels of anxiety and higher levels of self-compassion in response to critical feedback from an actor (Cardi et al., 2015).

A common limitation of these studies is that they have not measured changes in core eating disorder symptoms following CBM-I. Procedures for CBM-I in other mental health conditions, such as depression and anxiety disorders, seem to produce only a small effect on clinical symptoms (Krebs et al., 2018). A possible reason to explain the lack of generalization effects on clinical symptoms is that training materials are not systematically developed and validated within the target population. Most existing interpretation bias trainings in eating disorders have been adapted from those originally developed for people with anxiety disorders and have been validated in adults. For example, Cardi et al. (2015) adapted stimuli originally developed by Hirsch et al. (2009) which included scenarios covering common worry topics, and tested these in a feasibility study involving a sample of 28 females with anorexia nervosa. Furthermore, these materials were adapted by researchers without the involvement of key stakeholders including patients, health professionals, and carers. These factors limit the ecological validity of the training for the target population within today’s social context (Hughes et al., 2016). Based on this hypothesis, this study involved the development of stimuli for a novel CBM-I training for adolescents with eating disorders, and piloting the face validity of these stimuli. The three aims were:

1. To conduct a critical review of the literature on interpretation bias assessment and training towards social stimuli in eating disorders.
2. To conduct focus groups with adolescents with eating disorders, carers and professionals to identify salient themes around the topic of social rejection and generate scenarios (interpretation bias training stimuli) reflecting those themes.
3. To pilot the face validity of these materials, focusing on two key aspects (age-relevance and emotional salience) in the target population (adolescents with eating disorders).

## Materials and Methods

### Aim 1

The literature on interpretation bias towards social stimuli in people with eating disorders or studies involving community samples and included a measurement of eating disorder symptoms were reviewed. An online literature search was conducted using EMBASE, MEDLINE, PsycINFO, Web of Science, and PubMed from database inception – October 2019. Search terms included “interpretation bias” or “biased interpretation” in combination with “anorexia nervosa” or “bulimia nervosa” or “eating disorder” in the Title/Abstract or full-text fields. Publications were included if (1) they were published in a peer-reviewed journal and written in English, (2) used an assessment task to measure interpretation bias towards social stimuli or a cognitive bias modification training to reduce negative interpretation bias towards social information and (3) included a sample of children, adolescents or adults with a diagnosis of an eating disorder or included a community sample and a measurement of eating disorder symptoms (Table 1). The materials used for the assessment or training task used within each study are summarized in Table 1.

**TABLE 1:** Literature review of materials used for interpretation bias assessment or training.

**Materials (Authors)**	**Population and age group**	**Description of Material**	**Development of Material**
Summers and Cougle, 2018	Undergraduate female psychology students and women from the community (*N* = 41) with elevated symptoms of Body Dysmorphic Disorder	135 scenarios describing situations involving the risk of social evaluation or exposure to own appearance	Developed by the researchers.
Cardi et al., 2015	Women with anorexia nervosa (*N* = 28)	134 scenarios describing ambiguous situations involving the risk of social rejection	Adapted from Huppert et al., 2007; Hirsch et al., 2009; Hayes et al., 2010
Cardi et al., 2017	Women with anorexia nervosa (*n* = 35) and healthy controls (*n* = 30)	12 scenarios describing ambiguous situations involving the risk of social rejection	Adapted from Huppert et al., 2007; Hayes et al., 2010 Research team (4 individuals) independently chose subgroup of sentences with greatest potential of being interpreted in positive or negative way.
Turton et al., 2018	Women with anorexia nervosa (*N* = 55)	110 scenarios describing ambiguous situations involving the risk of social rejection	Adapted from Huppert et al., 2007; Hirsch et al., 2009; Hayes et al., 2010; Cardi et al., 2015
Matheson et al., 2018	Female undergraduates (*N* = 123)	87 scenarios describing appearance-relevant ambiguous social scenarios	Training stimuli were developed by the researchers and informed by appearance-based feedback and rejection sensitivity scales (Tantleff-Dunn et al., 1995; Altabe et al., 2004; Park et al., 2010; Park, 2013). These were rated by women in a pilot study for relatedness to appearance and affective valence.
Cardi et al., 2019	Adolescent girls with anorexia nervosa (*N* = 24)	112 scenarios describing ambiguous situations involving the risk of social rejection	Adapted from Cardi et al., 2015 in collaboration with five adolescents with anorexia nervosa receiving inpatient care.

### Aim 2

In order to address the main research question, “What situations are likely to trigger fear of being rejected/left out/excluded in adolescents with eating disorders?”, patients with eating disorders, carers and health professionals were invited to participate in separate, live online group forums which were themed around the topic of social rejection. Participants were recruited via opportunity sampling from a specialist eating disorder intensive treatment service and from a departmental database of patients who had previously participated in research and had opted to be contacted about future studies. Participants were required to be fluent in English and have no severe medical or psychiatric comorbidities in order to take part. Patients’ eligibility was assessed by the researcher and the eating disorder diagnosis was confirmed by a Consultant Psychiatrist based on DSM-5 criteria or self-reported by the patient (in three cases). A standard topic guide including questions related to the topic of social rejection was developed (Table 2) and the wording was adapted for patients, carers and health professionals. Two separate groups were held for the professionals to accommodate the availability of participants. The forums were hosted on a bespoke research platform that developed for another study running in the department (created by https://www.mindwaveventures.com/ and funded by the National Institute for Health Research - Health Technology Assessment). The forums were live, text-based groups accessible to participants only. All participants were given a participant screen name (e.g., Participant 1) in order to anonymise them. The researcher led the groups by posting open questions and providing participants with time to respond to each question. Each group lasted for one hour. Ambiguous scenarios related to the risk of rejection from others were then generated with consideration of those previously adapted for adolescents with eating disorders (Cardi et al., 2019) and the data derived from the focus groups. Each scenario consisted of a hypothetical ambiguous social situation which was open to the young person for interpretation (e.g., “It is a classmate’s birthday and your good friend brings in a cake. You aren’t offered a slice and feel awkward. Later you speak to your friend about how this made you feel and they…”).

**TABLE 2:** Topic guide for focus groups.

What situations can you think of that would involve the chance that you would be rejected by others? This can include being judged, criticized, or left out of a group.
Can you think of some examples of the sorts of things that you have avoided saying or doing in front of others, because you were worried about how others might react? E.g. worried about whether they would judge or criticize you.
What happened the last time you felt excluded / left out of a group?
What happened the last time you avoided a situation because you thought you might be rejected by others?
What would help you to feel more confident (i.e., less fearful or avoidant) in social situations (what could protect you from being rejected)?
How could others (i.e., treatment team, family) help you to feel more confident (less fearful/avoidant) in social situations?


### Aim 3

**FIGURE 1:**
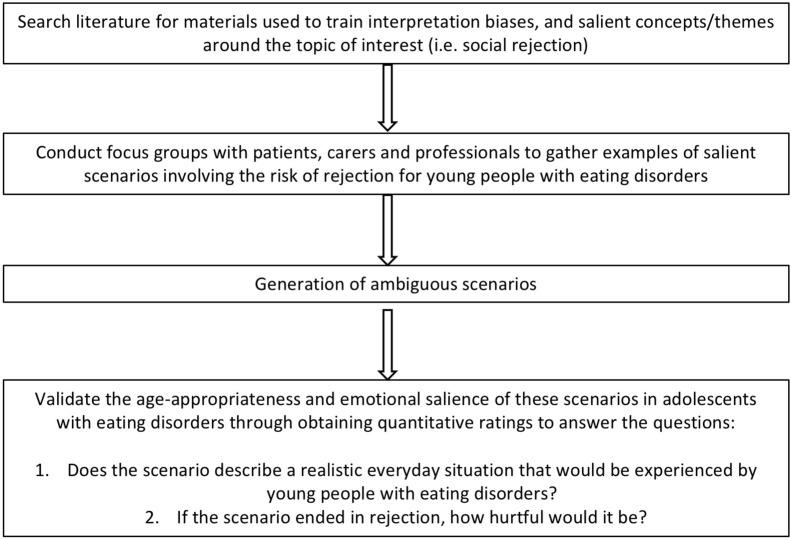
Flow diagram for interpretation bias stimuli development.

Quantitative methods were used to assess the degree to which the scenarios reflected realistic concerns that were both age appropriate and emotionally salient to young people with eating disorders (see Figure 1). Girls with eating disorders (*N* = 27) aged 13–17 who participated in the focus groups and girls attending an intensive treatment program who did not participate in the focus groups were invited to rate the scenarios (at this time there were no boys available in the service to rate the scenarios). Participants were provided with paper forms over email, or in person at their treatment center. Each paper form contained a proportion of the scenarios depending on the participants’ time availability, and two 5-point Likert-scales which were used to measure the age-relevance and emotional salience of each scenario. The scales ranged from 0 (not at all realistic) to 5 (very realistic) and 0 (not at all hurtful) to 5 (very hurtful). After discussion with the research team, the matrix (Table 3) was developed to decide whether to include, modify or exclude each of the scenarios. The scenarios that were considered both realistic and hurtful (scenarios scoring 3 or above on both scales) were included in the final selection of scenarios. The data that support the findings of this study are openly available in [repository name e.g., “figshare”] at, reference number [reference number].

**TABLE 3:** Ratings matrix to determine which scenarios would be included, modified or excluded.

**Age relevance**	**Emotional salience**		
	
**Ratings**	**1–2**	**3**	**4–5**
1–2	Exclude	Exclude	Exclude
3	Exclude	Revise and include	Revise and include
4–5	Exclude	Revise and include	Include

## Results

### Results of Aim 1

Six papers meeting the inclusion criteria were identified and reviewed (see Table 1). Five studies included adults (*N* = 312) aged 18–65 and one study included adolescents (*N* = 24) aged 14–18. With the exception of Cardi et al. (2019) that involved adolescents with lived experience of eating disorders in the process of adapting materials for an interpretation bias modification training, all five studies included stimuli adapted by the researchers that were originally designed for other populations. This presents missed opportunities to tap into concerns around social rejection that may be relevant to people with eating disorders in particular, such as fears around being rejected due to the physical or behavioral symptoms that are specific to anorexia nervosa, and to explore concerns that may be predominantly relevant to adolescents with these conditions.

### Results of Aim 2

Five focus groups were held in total. The patient group consisted of eight young people (seven females, one male) aged 13–17 (M = 15.50, SD = 1.22) with anorexia nervosa (*n* = 6) or bulimia nervosa (*n* = 2). A separate focus group was held for carers (*n* = 7) who were females aged 40–58 (M = 50.33, SD = 5.59). Only one of the carer participants was related to one of the patient participants in the study. Two separate groups were held for health professionals who were females aged 23–60 (M = 35.67, SD = 12.02) working within specialist eating disorder services. All participants were living in the United Kingdom. One group included two psychiatrists and two psychologists, and one group included a consultant psychologist, an assistant psychologist and a mental health support worker.

Data collected from the focus group transcripts were analyzed using content analysis to identify recurrent examples of social rejection scenarios (Table 4). Two researchers manually coded the data and used the qualitative data analysis software NVivo (Version 13). First the researchers familiarized themselves with the data through repeatedly reading the text of all transcripts and identifying initial codes (i.e., sentence by sentence coding). The researchers then categorized the examples of social rejection described by patients, carers and health professionals into themes, including virtual rejection/exclusion, rejection associated with an aspect of the eating disorder, rejection triggered by ambiguous/benign comments or behaviors from others and rejection perceived when confiding in others (see Table 4).

**TABLE 4:** Themes and sub-themes derived from focus groups with patients, carers and professionals.

**Theme**	**Patients**	**Carers**	**Professionals**
Virtual rejection/exclusion	“oh yeah and like posting photos and seeing how many likes/followers you have seems risky” “or you might worry they think you look fat/might not comment on you being skinny” “after I was discharged from hospital one of my friends always talked about the chat with my other friends who I walked to school with knowing full well I wasn’t in the chat”	“with social media, my daughter gets very concerned if she doesn’t get immediate responses to messages as she thinks she is being ignored. She doesn’t post much and in fact took down most of her social media during her GCSE’s.” “the “likes” on social media seems to be the way to judge popularity. My daughter used to spend ages trying to take a good photo to put on Instagram and would continuously check the likes and comments” “Yes online media is a nightmare for someone who has major anxieties, low self- esteem etc., Constantly looking for the number of ‘likes’ - spending hours over which picture to post and which caption she should add in case it is laughed at/ridiculed by others”	“seeing friends message each other on social media and not being included, seeing photos of friends hanging out at parties, etc., that they haven’t been invited to” “Not invited to parties and seeing this on the social media or being “blanked” by their peers, getting silent treatment” “friends taking a while to reply to messages” “I have seen young people feel so uncontrollably anxious that they have had panic attacks because friends have not texted them back and they are sure their entire social network has been lost.” “they would essentially go into a full-blown panic and experience severe anxiety if they did not receive enough likes on their post or if people unfriended them or unfollowed them or did not follow them back”
Rejection associated with an aspect of the eating disorder	“they go out for a meal but don’t invite you I have also had it happen when they go for a meal but don’t invite you or your best friend either because they don’t want you to feel left out” “Exactly, and sometimes it’s not just meals but on someone’s birthday they might bring in food but won’t ask if you want some or something along those lines” “Another example I had was my close friends all went out to play tennis and didn’t even ask as they assumed, I couldn’t do it”	“family gatherings with lots of food and expectations were also a problem for us at the time. Harmless statements such as “you’re up for dessert seconds” (dessert was never a problem) could send her into a tailspin” “Oh, the stresses of what to wear! it could take hours for her to get ready and the smallest of comments could send her back upstairs to get changed again!” “at present she is isolated and avoiding situations where she feels people are looking at her and judging (to be fair people are looking at her. her BMI is 13 and she looks ill, so it is natural for people to look). Sometimes she has felt excluded so has tried to be popular by dressing up (when she at a healthier weight she is stunning) and getting the boys attention.”	“or friends going out for lunch/to a party/to dinner and not inviting them because of the food element” “some negative comments from others about how thin they are, particularly from boys in their school, things like ‘you might snap’ I have heard quite a few times maybe partly due to fear that their peers would not see them as having an eating disorder, or thinking that they are ‘getting better’ a person who wasn’t selected for their sports team because they really weren’t well enough and they really took it to heart - it seemed to really impact their (already low) self-esteem and it was like it was a personal slight against them rather than a reflection of how poorly they were” “there was a lot of assumed rejection or criticism relating to competitiveness - so some young people might sit on the edge of their seat or stand for long periods of time so others would not judge them as lazy or not struggling during meal times, the food would be consumed very slowly, kind of like the opposite of a race”
Rejection triggered by ambiguous/benign comments or behaviors from others	“also people with eating disorders might misinterpret situations and react more negatively compared to people without who might not be upset by it” “Yes I agree with p4 definitely over think the reason you weren’t invited.” “Yes definitely organizing something with friends because sometimes if they don’t reply you might think they don’t want to come etc”	“I agree that you have to be very careful what words you use. A friend of mine said my daughter looked really well once and my daughter took that as she had put on too much weight.” “yesterday I commented to my daughter that her eyes looked brighter and she didn’t look so dehydrated. This translated into her mind as she looks like she has put on weight and looking healthier. she promptly shouted at me that I am triggering and went upstairs to weigh herself” “our daughter was constantly saying that teachers at school gave her “dirty looks” and was convinced that one subject teacher did not want her to study a subject at A	“Yeah, you look well doesn’t go down well!” “it is almost like any situation could make them feel rejected depending on even subtle reactions of the people they are with finding friends at lunch times and people not looking up straight away to say hello, or not moving over for them to sit in the group comfortably; peers finishing their conversations with other peers before greeting them; peers not really looking at them when they are talking in a group” people not asking them how they are (yet being asked also causes anxiety); people not making the effort to speak to them first; other ones might include seeing peers whispering and the young people believing they are
		Level. I had to ask the School to confirm if that was the case and they said absolutely not and that the teacher had no concerns over her ability”	speaking about them in a negative way, which might make them withdraw from those individuals.” “a young person could pay a compliment to another young person about their dress, and then would seek reassurance from a staff member that they have not just made the other young people feel bad/negative about their looks (if that makes sense) - so a lot of second guessing and worrying about what they say and do - and I guess worrying that this would lead to being socially rejected”
Rejection perceived when confiding in others	“when you tell a friend or someone else about the eating disorder and they don’t believe you/dismiss it” “I haven’t told anyone about my problem sometimes it feels to daunting to confront them about it, so sometimes I just leave it. I wouldn’t tell them how I felt because I would fear being rejected yet again by them.” “sometimes I try to hide my true feelings from my family and my other friends, and I just hide away”	“my daughter refused to tell anyone what was wrong with her for many years…apart from close family. her friends, etc., probably guessed but never asked. As she got older she told a few people. their reactions varied. they usually promised to be there for her and texted her for a week or so after but then that was it. sometimes they told other people which broke her trust. I know many other sufferers who are very open about their illness so I guess that varies for years didn’t tell anyone, even when she had long stays as in-patient. now she is 17 and told a few people, but still dificult to be completely up front with people”	“telling even close friends how they really feel” “I have had a few patients who have felt very anxious about letting people know about their eating disorder - understandable- and I guess at least part of that is due to fear of possible rejection, so they end up saying “I’m fine” even to close friends even though they’re not if someone is unpleasant or they perceive to have been unpleasant, then they will tell me that they do not know what to say, or only later they will think of something to say, or they will take it out on themselves (self-harm).” “it might be helpful to explicitly provide them with the tools to deal with these situations, e.g., workshops on how to safely confront someone or how to discuss sensitive topics with your peers if you think they are angry with you - basically like survival skills sessions for sensitivity to rejection”

### Results of Aim 3

Following Aims 1 and 2, 339 scenarios were generated by the research team. Each patient rated a minimum of 19 and a maximum of 264 scenarios depending on their time availability. The scenarios were then categorized using the ratings matrix (Table 3). One-hundred and sixty-six (49%) scenarios were rated high [scoring 4 or 5 on both age-relevance (M = 4.28, SD = 0.71) and emotional salience (M = 4.18, SD = 0.76)] and were automatically included in the final set of stimuli. Thirty-eight (11%) scenarios were rated as low (1 or 2) on one or both measures by at least one patient and automatically excluded. One-hundred thirty-five (40%) scenarios were rated as neutral (3) on both aspects and were then revised to increase their age-relevance and emotional salience by the researchers, so that they could be included in the final set of scenarios. Revisions to the neutral scenarios were made by taking aspects from the scenarios rated as high or feedback from young people rating the scenarios and applying them to the neutral scenario. For example, “Your friends are looking at a magazine over lunch at school. One of them turns the page and makes a comment about a thin model, so you ask if they can change the page” was changed to “your friends are looking at Instagram during the school lunchbreak. One of them starts looking at an Instagram feed and makes comments about a thin model, so you ask if they can look at a different account.”

The final set of stimuli consisted of 301 scenarios. All scenarios, with ratings for each of them presented separately are openly available in [repository name e.g., “figshare”] at, reference number [reference number].

## Discussion

This study followed the evidence-based practice framework and a systematic process of adapting experimental paradigms to specific populations (Hughes et al., 2016) to develop and validate stimuli for a cognitive bias modification training to reduce interpretation bias towards negative social stimuli in young people with eating disorders. The first aim was to review existing studies that have used cognitive bias assessment or modification procedures to target an interpretation bias towards negative social stimuli in people with eating disorders. The majority of identified studies included only adults and the majority of materials used within the training protocols had been adapted by the researchers from those originally developed for people with anxiety disorders (Cardi et al., 2015, 2017; Summers and Cougle, 2018; Turton et al., 2018). The second aim was to generate real-life examples of scenarios in which individuals with eating disorders feel exposed to social rejection or exclusion. Focus group discussions were held with young people with lived experience of eating disorders, carers and healthcare professionals with experience working with young people with eating disorders in specialist treatment services. Four key themes were identified from the focus groups including (1) virtual rejection/exclusion (2) rejection associated with an aspect of the eating disorder (3) rejection triggered by ambiguous/benign comments or behaviors of others and (4) rejection perceived when confiding in others. The final aim was to obtain quantitative ratings from young people with eating disorders concerning the age-relevance and emotional salience of the scenarios, and to make final adjustments to the scenarios based on this feedback. A total of 301 scenarios were included in the final stimulus set.

The findings from the focus groups with patients, carers and healthcare professionals in this study support the literature on social functioning in young people with eating disorders (Caglar-Nazali et al., 2014; Cardi et al., 2018a,b). All groups agreed that patients perceive rejection when exposed to ambiguous/benign comments or behaviors from others, such as over-thinking the reason for not being invited to a party or receiving a compliment on their physical appearance. These observations corroborate findings from an earlier qualitative study in which young people with anorexia nervosa described a heightened sensitivity to any form of perceived criticism (Patel et al., 2016) and recent quantitative data, which demonstrated that young people with anorexia nervosa on average produced more negative than benign interpretations of ambiguous social scenarios involving the risk of rejection (Cardi et al., 2019).

Participants also recalled examples of illness-related rejection experiences, such as being excluded from arrangements to have meals out with friends or not being offered food in social contexts. These findings support other qualitative accounts from young people with anorexia nervosa who have described the impact that their eating disorder and treatment regime had on their social functioning (Lindstedt et al., 2018). Furthermore, the risk of rejection perceived by patients when confiding in others, for example a reluctance to disclose their illness to friends due to a fear of being dismissed or disbelieved, supports the interpersonal model of eating disorders which posits that some problems with social functioning in people with eating disorders may originate in part from maladaptive personality traits, such as the tendency to avoid expressing feelings and the tendency towards interpersonal distrust and negative interactions with others (Arcelus et al., 2013).

Participants referred to several examples of virtual rejection, and over-reactions to this experience such as full-blown panic or severe anxiety if a friend did not reply to their message, or if they did not receive enough likes on their social media posts. These findings are novel in the context of the existing literature on interpretation bias, which has lacked the consideration of exposure to rejection in a virtual environment. However, the findings are in line with what is known about the role of online social interactions in eating disorders. For example, one study found that girls and women with a lifetime diagnosis of an eating disorder reported poorer mood after posting or commenting online, a greater frequency of social comparison, and a greater use of online forums and blogs with more focus on eating disorder-related issues in comparison to a group of age-matched controls (Bachner-Melman et al., 2018). In both groups, these online behaviors correlated with eating disorder symptoms and general psychological health. In another study, the frequency of Facebook use was associated with greater disordered eating, and maintenance of weight/shape concerns and state anxiety compared to an alternative online activity (Mabe et al., 2014). Together, these findings suggest that negative interpretations of social cues online may contribute to feelings of rejection and trigger eating disorder symptoms.

### Strengths and Limitations

The main strength of this study is that it is the first study to use a systematic approach to the development of stimuli for an interpretation bias training intervention for people with eating disorders. Other strengths include the involvement of different parties, including patients, carers and professionals (Kimber et al., 2019), to identify the type and content of social situations that trigger social evaluative concerns in adolescents with eating disorders, and the incorporation of both qualitative and quantitative methods in the development and validation of the materials. Furthermore, the large pool of materials developed (*N* = 301) will be made publicly available, in line with the open science framework and may be used for multi-session interpretation bias training protocols, which have shown advantages over single-session trainings in terms of training efficacy on interpretation bias change (Menne-Lothmann et al., 2014; Cristea et al., 2015; Turton et al., 2018). One limitation of this study was that due to the large number of scenarios and limited availability of patients, quantitative feedback was obtained only from a subgroup of adolescents with eating disorders (girls aged 14–18) and might not be generalisable to other groups of individuals, particularly younger girls or boys who may differ in their experiences of social rejection. Furthermore, some scenarios only had one rating from one participant. The scenarios rated by participants as neutral (*n* = 135) were revised further by the researchers to increase their age-relevance and emotional salience so that they could be included in the final set of scenarios. Although these adapted neutral scenarios were not included in the pilot study, they will be included in a proceeding study, investigating the feasibility and clinical effectiveness of multi-session cognitive bias modification training.

### Clinical Implications

There is increasing interest in the use of treatment enhancers in eating disorders due to their potential to improve clinical outcomes. In the United Kingdom, the National Institute for Health and Care Excellence (NICE) guidelines recommend Family Based Treatment as the first-line treatment for adolescents with eating disorders (National Institute for Health and Care Excellence (NICE), 2017). This therapy aims to improve nutrition and mostly focuses on providing information and support to carers to feed their children (Le Grange and Eisler, 2009; Lock and Le Grange, 2019). Whilst this is the most effective treatment available according to the current evidence base, approximately 20% patients offered Family Based Treatment drop-out (Dejong et al., 2012), between 33% and 42% reach remission by the end of treatment (Lock et al., 2010; Agras et al., 2014), and 40% of patients struggle with significant ongoing psychological distress after treatment (Lock et al., 2006; Wufong et al., 2019). CBM could provide a useful ‘treatment enhancer’ by increasing sensitivity to positive social feedback and reducing sensitivity to social criticism from family and peers, and the online nature of the training may appeal to the younger population.

The large set of stimuli described here has been developed and validated by girls with eating disorders. Multi-session studies combined with follow-up assessments allow for an investigation of the acceptability and effectiveness of the training in the long term. In doing so researchers should consider strategies for facilitating participant engagement with the training over time (Zhang et al., 2018) and to assess whether changes observed in interpretation biases are associated with changes in social perceptions and behaviors, as well as key clinical variables. It will also be important to explore potential pathways through which this training can be related to improvements in symptoms, such as through strengthening responsiveness to social acceptance or support. The training may also provide benefits to individuals at ‘high risk’ of psychopathology through boosting resilience to the risk of social rejection/exclusion.

### Conclusion

To date, this is the first study to use a systematic approach to the development of a cognitive bias training targeting an interpretation bias towards negative social stimuli in young people with eating disorders. These materials will be made available to aid researchers in conducting experimental studies to assess the acceptability and clinical effectiveness of multi-session cognitive bias modification training protocols in young people with eating disorders.

## Data Availability Statement

The datasets generated for this study are available on request to the corresponding author.

## Ethics Statement

This study was reviewed and approved by London Riverside – Research Ethics Committee. Written informed consent to participate in this study was provided by the participants’ or their legal guardian/next of kin.

## Author Contributions

KR, EW, MS, AH, and VC contributed to the study design. KR and EW performed the data collection and analysis which was supervised by VC. KR, EW, and VC wrote the manuscript with input from MS and AH.

## Conflict of Interest

The authors declare that the research was conducted in the absence of any commercial or financial relationships that could be construed as a potential conflict of interest.
